# Majoring in nutrition influences BMI of female college students

**DOI:** 10.1017/jns.2015.24

**Published:** 2016-02-08

**Authors:** Mee Young Hong, Tahirih L. Shepanski, Jaclyn B. Gaylis

**Affiliations:** School of Exercise and Nutritional Sciences, San Diego State University, San Diego, CA 92182, USA

**Keywords:** College students, Majoring in nutrition, BMI, Dietary habits, Health behaviour, NMs, nutrition major students, OMs, non-nutrition major students, SDSU, San Diego State University

## Abstract

Maintaining healthy eating habits in college is challenging. Interventions focused on nutrition education can assist in reversing these trends of poor eating habits among college students. The purpose of the study was to identify factors affecting the dietary habits, food choices and BMI of college females majoring in nutrition (NMs) compared with non-nutrition majors (OMs). A questionnaire-based cross-sectional survey study of dietary behaviour and food frequency of 202 college females was conducted at San Diego State University. Data were analysed by using *t* tests, χ^2^ tests and regression analysis in SPSS. NMs exhibited a lower BMI than OMs (*P* < 0·01); however, BMI values for both groups were within a healthy range. Interestingly, 3 % of NMs had a BMI in the range of overweight or obese; however, prevalence was three times higher for OMs, being 9·2 %. A healthier meal option was the most influential factor in NMs’ meal choices whereas convenience and weight control were influential factors in OMs’ meal choices. Most NMs read nutrition labels and reported that this affects their food choices. NMs exercised longer than OMs in the <120 min/week category. Exercise affected healthy meal conception in NMs only (*P* < 0·001). Taking dietary supplements influenced healthy meal awareness in OMs only (*P* < 0·05). University-level nutrition education is strongly associated with healthier eating habits and superior food choices among young adult females. More regular meal patterns, healthier snack choice and adherence to dietary guidelines may contribute to the lower BMI values observed among NMs compared with OMs.

Nutritional intake during young adulthood supports physical health, affects risk for future disease and plays a role in the prevention of excess weight gain. The lack of variety in college students’ food choices were revealed in data from the 2012 American College Health Association-National College Health Assessment (ACHA-NCHA), where only 5·3 % consumed the recommended daily intake of fruit and vegetables and most ate diets excessive in fat^(^^1^^,^^2^^)^. Dietary intake low in fruits and vegetables while high in refined carbohydrates and fats is closely linked to one's BMI and directly correlates with the risk of chronic disease in late adulthood^(^^3^^,^^4^^)^.

Statistics from San Diego State University's (SDSU) campus ‘Quick Serve Restaurants’ demonstrate students’ high consumption of unhealthy food and low consumption of healthier items. In January 2013, 8607 out of 38 495 students purchased à la carte French fries; that increased by 11 % to 12 817 total purchases in 2014 at SDSU's Towers Kitchen^(^^5^^)^. The sale of vegetable dishes dropped from 1580 in 2013 to 666 in 2014^(^^5^^)^. Given that poor dietary intake is one of the leading modifiable contributors to mortality, nutrition promotion among developing adults is an important focal area for research^(^^6^^)^.

National data also reveal that physical activity levels drop dramatically between junior high school and college graduation^(^^7^^)^. According to the ACHA-NCHA, only 19·5 % of college students engage in physical activity five or more days per week, with 25·2 % reporting zero physical activity in the last 7 d^(^^2^^)^. Additionally, in this demographic, there is a higher prevalence of dieting behaviours among college women who avoid gaining body fat, have lower self-esteem, and are preoccupied with body size and shape^(^^8^^)^.

Research shows that college students gain an average of 3·5 kg during their freshman year^(^^9^^)^. In order to reverse unhealthy eating habits that may lead to obesity, researchers have used nutrition education interventions among college students^(^^4^^)^. When comparing the BMI of freshmen that took a nutrition class *v.* those who did not, students with a high BMI who did not enrol in the nutrition class gained an average of 15 to 20 pounds (7 to 9 kg) over the 16-month period^(^^10^^)^.

Nutrition education during college years is vital in order to teach college students how to make healthy dietary and lifestyle choices which may affect their overall health and wellbeing.

Nutrition majors (NMs) not only have education on nutrition, food choices, dietary behaviour and diseases but also food preparation and exercise physiology. It is often assumed that NMs make better food choices and lead healthy lifestyles. However, the effectiveness of college nutrition courses on the habits of NMs in correlation with students’ BMI, snack choices, food habits and exercise patterns has not been well investigated. The objective of this study was to identify factors that affect the dietary habits and food choices of NMs and non-nutrition major students (OMs). We hypothesised that this formal education in nutrition would positively affect behaviours such as food choice and meal decisions, exercise and sleep patterns, and a healthy body image, which would demonstrate a measurable clinical outcome of a lower BMI in NMs compared with their non-biological-science peers.

## Experimental methods

### Subjects and environment

The study protocol was approved by the Institutional Review Board for research involving human subjects. The objective of this study was to identify factors affecting the dietary habits and food choices of NM compared with OM college female students. A total of 202 subjects (101 female NMs and 101 female OMs) volunteered to complete a survey. Inclusion criteria for a nutrition student was a course major in foods and nutrition while inclusion criteria for a non-nutrition student was any non-biological science or non-healthcare major such as language arts, business, psychology, etc. Exclusion criteria for both groups included: (a) biological science-based majors such as biology, chemistry and nursing as well as public health; (b) foreign exchange students or persons whose residency in the USA was less than 3 years; and (c) male students.

Surveys were completed by NMs in Exercise and Nutritional Science classes while OMs completed surveys in common areas on campus such as bookstores, residence halls and the library. Participants were provided an incentive equivalent to less than 1 US dollar after completing the survey. All participants were 18 years of age or older and provided informed consent at the time of the survey.

### Questionnaires

Questions regarding demographics consisted of age, height, weight, ethnicity, birthplace and duration in the USA. Respondents self-reported their anthropometric measurements. BMI was calculated as weight in kg divided by the square of height in m. The BMI of the subjects were compared with standards for BMI categorisation: <18·5 kg/m^2^ = underweight; 18·5 to 24·9 kg/m^2^ = normal; ≥25·0 kg/m^2^ = overweight/obese. Lifestyle pattern questions included topics such as dietary habits, health concerns, sleep patterns, exercise regimens, stress levels, body image and potential disordered eating. Subjects were also asked questions regarding nutrition knowledge such as nutritional labelling, sources of nutrition information and adherence to published dietary guidelines^(^^11^^)^. An FFQ asked how often subjects consumed certain food items: grains, starches, meats, fish, fruits, vegetables, dairy products and beverages including alcohol. The nine potential frequency choices ranged from three times per d to never^(^^12^^,^^13^^)^.

### Statistical analyses

Data were analysed using SPSS (version 20; IBM Inc.). Q–Q plots for each of the dependent variables were carried out to test normality. Student's *t* tests with a Bonferroni adjustment were used to analyse the age, weight, height, BMI and lifestyle questions with numerical values. We used χ^2^ tests to examine differences with categorical variables, such as food frequency, dietary intake and the majority of the lifestyle questions. Correlations among variables were tested using Pearson correlation or Spearman's *ρ* analysis. Regression analysis was conducted to identify factors affecting self-reported healthy meals. Data are presented as means and standard deviations and statistical significance was set at *P* ≤ 0·05.

## Results

### Demographics

Subjects included 101 NMs and 101 OMs and the participants’ mean age was 23 years. Based on the standards for BMI, 6 % of the subjects were underweight (BMI < 18·5 kg/m^2^), 88 % were normal weight (BMI 18·5 to 24·9 kg/m^2^) and 6 % were overweight/obese (BMI ≥ 25·0 kg/m^2^ (Table 1). BMI was significantly different between the two groups; NMs exhibited a lower BMI than OMs (*P* = 0·005). Only 3 % of NMs had a BMI in the range of overweight or obese, where it was three times higher being 9·2 % for OMs (Table 1). However, mean BMI values for both groups were within a healthy range. Of the subjects, 79 % identified their ethnicity as white, 9 % as Hispanic, 6 % as other, 4 % as Asian and 2 % as black (Table 1).

**Table 1.** Characteristics of participating female college students (Mean values and standard deviations or percentages)

	NMs (*n* 101)		OMs (*n* 101)	
	Mean	sd	Mean	sd
Age (years)	24·3	3·9	22·4	4·0
Weight (kg)	58·8	7·4	60·3	9·1
Height (cm)	166·4	6·6	166·0	7·5
BMI (kg/m^2^)*	21·2	2·0	21·3	3·1
BMI classification (%)†				
Underweight	5·9		6·1	
Normal	91·1		84·7	
Overweight/obese	3·0		9·2	
Ethnicity (%)				
White	77·2		81·2	
Black	2·0		2·0	
Hispanic	12·9		5·9	
Asian	1·0		5·9	
Other	6·9		5·0	

NMs, nutrition major students; OMs, non-nutrition major students.

* Mean values were significantly different between NMs and OMs (*P* < 0·05, *n* 202; non-paired *t* tests).

† BMI classification: <18·5 kg/m^2^ = underweight; 18·5 to 24·9 kg/m^2^ = normal; >25·0 kg/m^2^ = overweight/obese.

### Lifestyle habits and health practices

In this sample of female college students, 88 % were non-smokers and 73 % reported drinking alcohol one to three times per month (Table 2). There was no difference between NMs and OMs on smoking and drinking habits. Mean length of sleep was significantly different between groups (NMs = 7·2 (sd 1·1) h; OMs = 6·7 (sd 1·8) h; *P* = 0·001). Of the subjects, 69 % answered that they have sufficient sleep to recover from fatigue but 31 % feel not sufficient; 52 % of subjects reported feeling ‘slightly stressed’ while 44 % reported feeling ‘very stressed’. Overall, more than 90 % cited school and family as the reasons for stress.

**Table 2.** Female college students’ lifestyle habits and health practices (%)

	NMs (*n* 101)	OMs (*n* 101)	Overall
No smoking	91	84	88
Alcohol drinking	70	76	73
Sleep sufficient	69	68	69
Stress			
Slight	52	52	52
Very	45	52	44
Try to lose body weight	48	53	51
To improve health	45	40	42
To look better	52	53	53
How to control body weight			
Diet control*	20	11	16
Exercise*	35	58	47
Both*	40	22	31

NMs, nutrition major students; OMs, non-nutrition major students.

* NMs mainly changed their dietary and exercise habits while OMs mainly focused on enhancing their exercise (*P* < 0·05, *n* 202; χ^2^ test).

About 51 % of subjects had tried to lose weight (Table 2). Of the subjects, 53 % cited the reason for weight control ‘to look better’ while 42 % cited wanting ‘to improve health’. Both NMs and OMs focused on exercise and/or changing their dietary habits when trying to control their weight. However, NMs mainly changed their dietary and exercise habits while OMs mainly focused on enhancing their exercise (*P* < 0·05) (Table 2). There were no differences between NMs and OMs on the questions regarding disordered eating or potential eating disorder.

Of the subjects, 70 % reported exercising ‘regularly’ during the previous month. NMs reported exercising 3·24 d/week with a mean of 62 min/d (Table 3). OMs exercised 3·07 d/week with a mean of 58 min/d. No statistical difference was found between NMs and OMs on exercise frequency and duration. Yet, out of the NMs and OMs who exercise under the recommended 120 min/week, NMs recorded an average 26·1 min/week *v*. OMs at 7·2 min/week (*P* = 0·032) (Table 3).

**Table 3.** Female college students’ exercise frequency and duration (Mean values and standard deviations or percentages)

	NMs (*n* 101)		OMs (*n* 101)		Overall	
	Mean	sd	Mean	sd	Mean	sd
Exercise (d/week)	3·24	1·91	3·07	1·90	3·15	1·91
Exercise (min/d)	62·3	37·7	58·0	40·8	60·2	39·3
Exercise (min/week)	240	189	212	208	226	199
<120 min/week (min/week)*	26·1	37·6	7·2	16·6	16·7	26·9 (28)
Percentage	23		33		28	
120–210 min/week (min/week)	187·6	93·0 (33)	176·0	77·8	181·8	93·0
Percentage	33		26		30	
>210 min/week (min/week)	388·2	93·0	397·6	80·9	392·9	93·0
Percentage	44		41		42	

NMs, nutrition major students; OMs, non-nutrition major students.

* NMs recorded higher average exercise time compared with OMs in the category of <120 min/week (*P* < 0·05, *n* 202; non-paired *t* tests).

### Dietary habits and meal factors

**Fig. 1.**
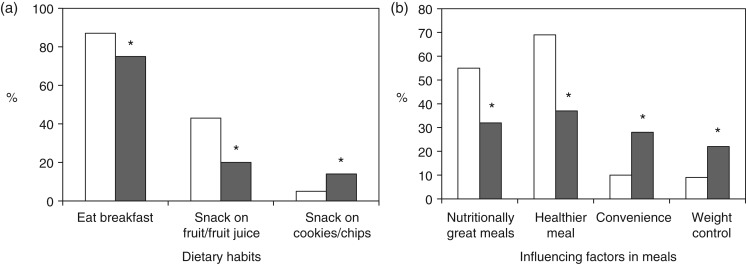
(a) Dietary habits on breakfast eating and snack choices between nutrition major students (NMs; □) and non-nutrition major students (OMs; ■). (b) Influencing factors in meals between NMs and OMs. * *P* < 0·05 (*n* 202; χ^2^ analysis). Values are percentages.

Of all subjects, 19 % reported skipping breakfast; however, 87 % of NMs reported eating breakfast in the 2 d prior compared with 75 % of OMs (*P* = 0·036) (Fig. 1(a)). In all, 55 % of all subjects snacked two times per d and 32 % of all subjects reported snacking on fruit and fruit juice (44 % NMs *v*. 20 % OMs) (*P* = 0·040); however, 14 % of OMs and 5 % of NMs reported snacking on cookies and chips (crisps) (*P* = 0·042). Fig. 1(b) presents significant differences between groups regarding influential factors in meals. Of NMs, 55 % reported nutritionally great meals *v*. 32 % of OMs reporting great meals (*P* < 0·001). A healthier meal option was the most influencing factor in 69 % of NMs’ meal choices while convenience (28 %) and weight control (22 %) as well as a healthier meal were the major influential factors in OMs’ meal choices (*P* < 0·05).

### Sources of nutrition knowledge

**Fig. 2.**
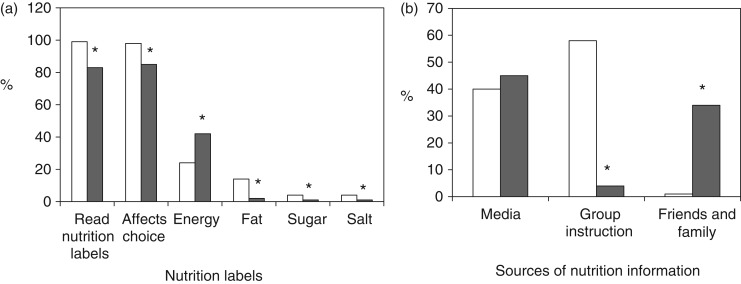
(a) Nutritional label reading between nutrition major students (NMs; □) and non-nutrition major students (OMs; ■). (b) Source of nutrition information between NMs and OMs. * *P* < 0·05 (*n* 202; χ^2^ analysis). Values are percentages.

Of NMs, 99 % reported that they read nutritional labels compared with 83 % of OMs (*P* < 0·001) (Fig. 2(a)). Additionally, 98 % of NMs *v*. 85 % of OMs reported that label reading ‘affects’ their food choices (*P* = 0·001). About 42 % of OMs read labels because they were interested in the total energy while only 24 % of NMs read labels for this reason (*P* = 0·021). NMs (14 %) reported being interested in the fat content on labels compared with only 2 % of OMs.

The media (television/Internet/news/books/magazines) were cited as a primary source of nutritional information for 45 % of OMs while 58 % of NMs cited group instruction (*P* < 0·001) (Fig. 2(b)). Additionally, 34 % of OMs received nutritional information from friends and family while only 1 % of NMs cited this source.

### Adherence to Dietary Guidelines for Americans

Subjects were presented with the Dietary Guidelines for Americans and were asked to determine whether they: (a) ‘practise well’, (b) ‘try to practise’, or (c) ‘don't practise’ each of the seven guidelines (Table 4). Results show that 60 % of NMs reported ‘well’ when eating a variety of foods, while 54 % of OMs reported that they ‘try’ (*P* < 0·001). When balancing food with physical activity to maintain or lose weight, 50 % of NMs reported doing this ‘well’ while 56 % of OMs reported that they ‘try’ (*P* = 0·041). More than 62 % of NMs reported choosing plenty of grains, vegetables and fruits and limited their dietary fat ‘well’ while 55 % of OMs reported that they ‘try’ (*P* < 0·001). About 37–39 % of NMs reported limiting their sugars and salt ‘well’ while 23–29 % of OMs reported that they ‘don't practise’ (*P* < 0·001). Last, 53 % of NMs reported drinking alcohol in moderation ‘well’ while 45 % of OMs reported that they ‘try’.

**Table 4.** Adherence to Dietary Guidelines for Americans (%)

Guideline	Practise?	NMs (*n* 101)	OMs (*n* 101)	P*
Eat a variety of foods	Well	60·4	36·6	
	Try	39·6	53·5	<0·001
	Don't	0·0	9·9	
Balance food and physical activity	Well	49·5	32·7	
	Try	44·6	56·4	0·041
	Don't	5·9	10·9	
Choose plenty of grains, vegetables and fruit	Well	73·0	37·6	
	Try	26·0	54·5	<0·001
	Don't	1·0	7·9	
Choose diet low in fat, saturated fat and cholesterol	Well	62·4	26·7	
	Try	34·7	59·4	<0·001
	Don't	3·0	13·9	
Choose diet moderate in sugars	Well	36·6	20·0	
	Try	58·4	57·0	<0·001
	Don't	5·0	23·0	
Choose diet moderate in salt and Na	Well	39·0	24·0	
	Try	49·0	47·0	0·005
	Don't	12·0	29·0	
Alcohol in moderation	Well	52·6	37·8	
	Try	33·0	44·9	0·110
	Don't	14·4	17·3	

NMs, nutrition major students; OMs, non-nutrition major students.

* NMs mainly changed their dietary and exercise habits while OMs mainly focused on enhancing their exercise (*P* < 0·05, *n* 202; χ^2^ test).

### Correlations

The correlation between certain health behaviours and BMI demonstrates that healthy habits tend to result in a lower BMI (Table 5). There was a strong positive correlation between thoughts of ‘feeling fat’ and a higher BMI (coefficient *r* 0·629; *P* *<* 0·001). Students with a higher BMI showed a higher tendency to lose body weight (*P* = 0·029). Nutrition label readers had a lower BMI compared with non-readers (*P* = 0·028) (Table 5). Those with a higher BMI also had a lower adherence rate in following the guidelines of eating more fruits and vegetables and consuming a low-fat diet (*P* < 0·05). People with a high BMI recorded low salad consumption (coefficient *r* – 0·216; *P* = 0·014) and high soda consumption (*P* = 0·050).

**Table 5.** Correlation of health behaviours with BMI

	Coefficient	P*
Feels fatty	0·629	<0·001
Try to lose weight	0·156	0·029
Choose grains, fruit and vegetables	−0·178	0·012
Choose low fat	−0·169	0·017
Salad consumption	−0·216	0·014
Soda consumption	0·173	0·050
Read nutrition label	−0·159	0·028

* Correlations using Spearman's *ρ* analyses between certain health behaviours and BMI demonstrate that healthy habits tend to result in a lower BMI (*P* ≤ 0·05, *n* 202).

Numerous factors were evaluated to determine whether NMs and OMs considered certain behaviours as having a positive influence and if those factors affected their perception of a healthy meal (Table 6). Both age and eating breakfast showed a significant difference overall, for both NMs and OMs, which positively affected the value of a healthy meal (*P* < 0·05). Adherence to the dietary guidelines, including eating grains, vegetables and fruit, is also significant for both NMs and OMs affecting their perception of a healthy meal (*P* < 0·05). Exercise affected healthy meal conception in NMs only (*P* < 0·001). Taking dietary supplements and reading nutrition labels influenced healthy meal awareness in OMs only (*P* < 0·05).

**Table 6.** Regression on factors affecting perception of a healthy meal* (Regression coefficients)

	NMs (*n* 101)		OMs (*n* 101)		Overall	
Variables	B	*P*	B	*P*	B	*P*
Age	0·056	<0·001	0·046	0·026	0·061	<0·001
Weight	−0·004	0·603	0·006	0·552	0·001	0·889
Height	0·005	0·609	0·016	0·163	0·012	0·124
BMI	0·033	0·284	0·033	0·923	0·024	0·245
Exercise time*	0·001	<0·001	0·000	0·494	0·001	0·003
Eating breakfast	0·448	0·013	0·698	<0·001	0·661	<0·001
Dietary supplement*	0·059	0·161	0·194	0·001	0·120	0·001
Read nutrition label*	0·690	0·267	0·542	0·007	0·711	0·001
Nutrition guideline						
Eat a variety of foods	0·469	<0·001	0·499	<0·001	0·599	<0·001
Balance food and physical activity	0·270	0·007	0·484	<0·001	0·433	<0·001
Grains, vegetables and fruit	0·511	<0·001	0·544	<0·001	0·605	<0·001
Fat, saturated fat and cholesterol	−0·330	0·003	−0·662	<0·001	−0·580	<0·001
Sugars	−0·350	0·001	−0·551	<0·001	−0·530	<0·001
Salt and Na	−0·237	0·010	−0·477	<0·001	−0·422	<0·001

NMs, nutrition major students; OMs, non-nutrition major students.

* Regression analyses indicated that there were some different factors affecting perception of a healthy meal between NMs and OMs (*P* < 0·05, *n* 202).

## Discussion

This study was designed to compare the dietary habits and food choices of female college NMs and OMs and their relationship with BMI. Our results indicate that NMs have lower BMI than OMs. A potential reason for this difference is that NMs are more focused on a healthy lifestyle and subsequently read labels more frequently. This practice has a positive effect on their food choices and there is evidence that ‘self-regulatory skills’, such as reading nutrition labels, positively affect food choices in college students^(^^14^^)^. These behaviours related to nutrition provide evidence that factors associated with dietary choices are logical places for knowledge-based interventions. Additionally, our results demonstrate that NMs are more likely to eat breakfast and consume healthier snack options than OMs. These healthier meal patterns may lead to the differences in BMI. This finding is consistent with other research that shows that young people who habitually eat breakfast have better body-weight management and reduced cardiometabolic risk factors compared with those who skip breakfast^(^^15^^,^^16^^)^. Breakfast skipping induces intense hunger and results in subsequent overeating in heavy, high-fat, high-carbohydrate meals and snacks later in the day. Studies have shown a significant relationship between breakfast skipping and low fruit and vegetable intake among female students^(^^17^^)^. Although there is some debate regarding an association with snacking, satiety and energy intake compensation in next meal^(^^18^^,^^19^^)^, healthy snack consumption should be beneficial to a quality diet and improved health^(^^20^^)^. Based on our findings, NMs, with additional formal nutrition, appear to choose healthier foods for their meals regardless of the times of day and frequencies at which they choose to eat.

NMs also look for total energy, energy from fat, Na and sugar content of the foods. Studies demonstrated that women who received energy information chose significantly lower-energy meals than women who did not receive energy information^(^^21^^)^. This practice combined with NMs reporting that they are more likely to ‘practise well’ six out of seven of the Dietary Guidelines for Americans may lead NMs to choose healthier foods for consumption. Reading the nutrition label in its entirety is essential to help people make the healthiest selection^(^^22^^)^. Since NMs study the science of how the human body responds to certain foods, they understand that choosing nutrient-dense foods over low-energy options benefits the body by providing it with nutrients and fuel. Due to their knowledge of nutrient density, NMs will make food choices based on the whole composition of the food, rather than solely on the total amount of energy.

Although the FFQ did not indicate significant differences in food choice between groups, portion sizes were not reported and portion sizes can make a difference in energy intake, which in excess correlates with weight gain^(^^23^^)^. Recognition of portion sizes and how to educate others on portion size is part of the university nutrition curriculum so it can be assumed that NMs share experience practising this health behaviour as well. The knowledge of portion control may correlate to the NMs’ lower BMI values compared with OMs’.

When evaluating certain factors that may influence one's perception of a healthy meal, factors such as age, consuming breakfast and adhering to nutrition guidelines are significant independent of being a NM. However, certain factors have varying effects on NMs and OMs. NMs associate exercising with a healthy meal, in contrast to OMs who do not. Moreover, OMs believed consuming a dietary supplement or reading nutrition labels improved the nutritional value of their meals, while NMs did not find these factors influential. This may be due to the fact that NMs already read nutrition labels and are more focused on regular diets rather than consuming dietary supplements. Discrete regression models to identify factors affecting the perception of a healthy meal between NMs *v*. OMs should be considered.

It is interesting to find that 70 % of NMs and OMs students exercised regularly (3 d/week, 60 min/d). Our study did not find significant differences between the NMs *v*. OMs with regards to exercise regularity, length, or intensity. However, the lower activity group was higher in OMs than NMs. Studies on freshman weight gain during the first year of college indicate that an increase in body weight is, in part, due to decreases in physical activity^(^^24^^,^^25^^)^. Future studies will include the comparison of exercise activity before, during and after college.

There is conflicting evidence whether nutrition education increases the risk of a potential eating disorder or disordered eating. Some studies show a correlation while other studies indicate that eating disorders or the risk for disordered eating behaviours are not more prevalent among nutrition students when compared with students from other courses^(^^26^^)^. The results from our study did not show a correlation between NMs and an increased risk of an eating disorder or disordered eating, as there were no differences between NMs and OMs. Future studies are necessary to investigate this possible association further.

Limitations of this study include the use of a convenience sample for surveying rather than a random sample. In addition, self-reported anthropometric measures were used. Some studies showed that disparities exist when using self-reported anthropometric measures; however, others have demonstrated high validity of self-reported height and weight^(^^27^^–^^29^^)^. We used BMI to assess relative health and fitness. Even though BMI is a useful way to classify body weight, it may not be the best indicator of health and fitness or chronic disease risk. Body composition, blood analyses and blood pressure may serve as additional indicators of health and fitness in future studies. While FFQ tools are a well-recognised method for dietary assessment in large-scale studies, it can be difficult to accurately estimate serving sizes and food intake. It remains to be determined whether or not the knowledge of portion control correlates with BMI.

## Implications for research and practice

Our study demonstrated that NMs were more likely to adhere to dietary guidelines, establish healthy meal patterns, and use nutritional information to choose healthier foods, which may contribute to their lower BMI values. As lower BMI is associated with decreased risk for chronic disease, our results add credence to the importance of health education for improving the lifelong health of college students. We have observed that nutrition education may improve the dietary and health behaviours of college students and may decrease obesity rates among young adults. Educators in nutrition and exercise play a vital role in helping students develop healthy habits in order to prevent obesity. NMs’ positive influence on campus for promoting these benefits may make a significant impact on those outside of their own major. We intend to further explore our findings in a larger cohort involving male and female college students in the future. It is of interest to investigate the different exercise habits and BMI of exercise physiology majors compared with NMs and OMs. Future studies will also include comparing how well students have maintained a healthy lifestyle post-graduation. We are interested to see if students from certain majors have implemented any healthy behaviours that they learned while in university.
